# Timing of Performing Endoscopic Retrograde Cholangiopancreatography and Inpatient Mortality in Acute Cholangitis: A Systematic Review and Meta-Analysis

**DOI:** 10.14309/ctg.0000000000000158

**Published:** 2020-03-18

**Authors:** Lijun Du, Mengsha Cen, Xia Zheng, Liang Luo, Ali Siddiqui, John J. Kim

**Affiliations:** 1 Department of Gastroenterology, Sir Run Run Shaw Hospital, School of Medicine, Zhejiang University, Hangzhou, China;; 2 Division of Gastroenterology & Hepatology, Loma Linda University Health, Loma Linda, California, USA.

## Abstract

Although early biliary drainage improves outcomes in patients with acute cholangitis, the optimal time to perform endoscopic retrograde cholangiopancreatography (ERCP) is controversial. Our aim was to evaluate the impact of timing of ERCP on mortality in hospitalized patients with acute cholangitis.

**METHODS:**

We searched PubMed, EMBASE, and The Cochrane Library (until February 2019) for studies evaluating the impact of timing of ERCP (<24, <48, and <72 hours from hospitalization) on outcomes in patients with acute cholangitis. The primary outcome was in-hospital mortality.

**RESULTS:**

Fourteen observational studies, including 84,063 patients (mean age = 66 ± 18), met the study criteria. The overall pooled in-hospital mortality with acute cholangitis was 1.9% (95% confidence interval [CI] 1.8%–7.6%), which increased to 4.3% (95% CI 1.8%–8.7%) when administrative database studies were excluded. In 9 studies, ERCP performed <24 compared with ≥24 hours decreased in-hospital mortality (odds ratio [OR] = 0.81, 95% CI 0.73–0.90; *I*^2^ = 0%). In 8 studies, ERCP performed <48 compared with ≥48 hours decreased in-hospital mortality (OR = 0.57, 95% CI 0.51–0.63; *I*^2^ = 0%). In 4 studies, ERCP performed <72 compared with ≥72 hours decreased in-hospital mortality (OR = 0.32, 95% CI 0.15–0.68; *I*^2^ = 0%). Furthermore, hospital stay was reduced in patients receiving ERCP <24 compared with ≥24 hours (mean difference [MD] = 3.2 days, 95% CI 2.3–4.1; *I*^2^ = 78%), <48 compared with ≥48 hours (MD = 3.6 days, 95% CI 2.1–5.1; *I*^2^ = 98%), and <72 compared with ≥72 hours (MD = 4.1 days, 95% CI 0.9–7.3; *I*^2^ = 63%).

**DISCUSSION:**

In observational studies, earlier ERCP performed in patients with acute cholangitis, even urgently performed <24 hours from presentation, was associated with reduced mortality. A randomized trial evaluating the impact of urgent ERCP on outcomes is needed.

## INTRODUCTION

Acute bacterial cholangitis is a gastrointestinal (GI) emergency associated with high mortality (1,2). Conservative management with antibiotics and intravenous fluid resuscitation without biliary drainage has a high risk of progression to sepsis. Early drainage to alleviate biliary obstruction, primarily by endoscopic retrograde cholangiopancreatography (ERCP), is the cornerstone management of acute cholangitis (3). Previous studies have demonstrated that the delayed performance of ERCP is associated with poor outcomes (4–6). Although the GI society guidelines recommend urgent ERCP in patients with severe acute cholangitis, the specific timing to perform ERCP is not clear and based on low level of evidence (7,8).

Although a number of observational studies have been conducted to evaluate the benefits of performing early ERCP in patients with acute cholangitis, most studies lacked sufficient number of patients to evaluate a meaningful impact on mortality (4–6). Furthermore, the optimal time of when to perform ERCP in patients with cholangitis is unclear. Therefore, we conducted a systematic review and meta-analysis of studies evaluating the impact of timing of ERCP on mortality in hospitalized patients with acute cholangitis.

## METHODS

### Search strategy and selection criteria

A systematic review of studies evaluating the impact of timing of ERCP on outcomes in patients with acute cholangitis was conducted using PubMed, EMBASE, and The Cochrane Library databases until February 2019. The following keywords or medical subject headings were used: “cholangitis,” “acute cholangitis,” “endoscopic retrograde cholangiopancreatography,” “ERCP,” “time to treatment,” “time,” “timing,” “initiation,” “early,” “earlier,” “late,” “delayed,” “accelerate,” “accelerated,” and “accelerating.” After the titles and abstracts of the identified articles were screened, full manuscripts of potentially relevant studies were retrieved to apply the study selection criteria. Studies were included if they met the following criteria: (1) hospitalized patients with acute cholangitis, (2) biliary drainage primarily by ERCP, and (3) compared outcomes as stratified by the time to ERCP from the initial hospital presentation (<12 vs ≥12 hours, <24 vs ≥24 hours, <48 vs ≥48 hours, and <72 vs ≥72 hours). When raw event numbers were unavailable, respective authors were contacted for additional data.

### Outcome of interest

The primary outcome was in-hospital mortality. When data on in-hospital mortality were not available, 30-day mortality was extracted to derive the primary outcome. The secondary outcomes included hospital stay, intensive care unit (ICU) admission, ICU stay, organ failure, and adverse events.

### Data extraction and assessment of quality of evidence

Data extraction was performed by 2 independent investigators (L.D. and M.C.) using a predefined data collection sheet, including study characteristics (author's name, publication year, country of study, total number of patients, mean age, study period, and study design) and main outcomes. When a discrepancy occurred between the investigators, the original publications were rereviewed until an agreement was achieved. The Newcastle–Ottawa Scale was used to grade the methodological quality of the observational studies (9). The scale consists of 3 items: selection, comparability, and ascertainment of outcome. From a score range of 1–9, studies with a higher score were considered to be higher quality.

### Statistical analysis

Demographic data were obtained by using pooled data from all included studies and expressed as mean ± SD or proportions with confidence interval (CI). Furthermore, pooled odds ratio (OR) with 95% CI was calculated for in-hospital mortality. The mean difference (MD) with 95% CI was calculated for hospital stay. Heterogeneity was assessed by the *I*^2^ statistic (*I*^2^ ≥50% indicating significant heterogeneity). In the absence of heterogeneity, a fixed-effect model analysis using the Mantel–Haenszel method for binary outcomes or inverse variance method for continuous outcomes was applied. Otherwise, a random-effect model analysis was used, and further sensitivity analysis was performed to evaluate the impact of a single study. The Begg and Egger tests were performed to evaluate the presence of publication bias. Two-sided *P* value <0.05 was considered significant. Stata 13.0 (StataCorp, College Station, TX) was used for all analyses.

## RESULTS

### Study selection

**Figure 1.**
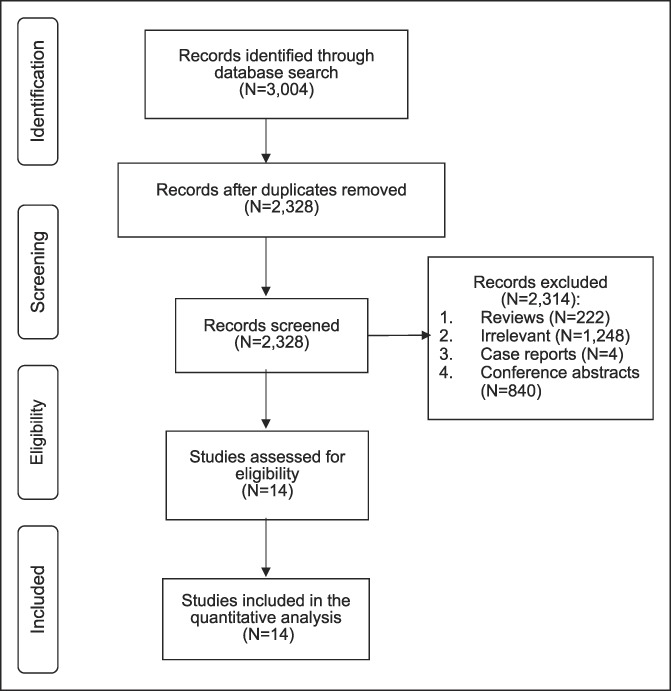
Flow diagram of study selection.

The search strategy retrieved 3,004 references. After removing duplicates, 2,990 were excluded for irrelevance based on title and abstract screening. Fourteen fully published manuscripts were selected for a full text analysis and included in the meta-analysis (Figure 1) (5,6,10–21). Two studies (14,15)used the same population to evaluate different outcomes. Authors were successfully contacted to obtain additional data on 3 studies (5,6,11).

Of the 14 studies, 10 were performed in the United States, 3 in Asia, and one in Europe (Table 1). Acute cholangitis was defined by the Tokyo Guidelines in 6 studies (10–12,18,19,21), the International Classification of Diseases, Ninth Revision, Clinical Modification codes in 3 studies (13,16,20), and other criteria in 5 studies (5,6,13,14,17). Eight studies stratified the severity of acute cholangitis by the Tokyo Guidelines (6,10–12,17–19,21). In 11 studies (5,6,10,12–14,16–20), the median proportion of patients with choledocholithiasis as the etiology of acute cholangitis was 57% (range 31%–100%). Five studies (5,6,10,16,19) provided data on the proportion of patients with concomitant acute pancreatitis (4.0%–14.6%) and 2 studies (5,14)on the proportion who received a liver transplant (1.5% and 2.3%).

**Table 1.**
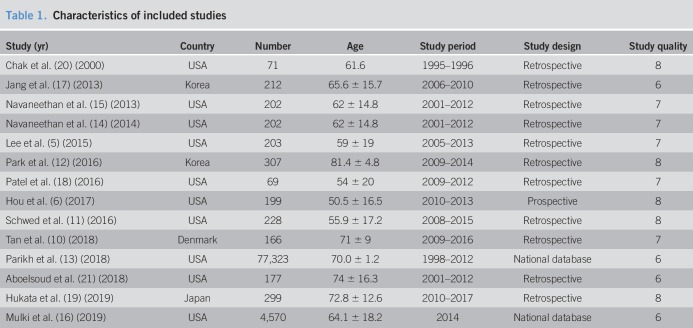
Characteristics of included studies

All patients received ERCP in 9 studies, and most patients received ERCP in 5 studies (61%–97%) (11–13,20,21). Outcomes for patients receiving ERCP and/or percutaneous drainage were not separately provided in 2 studies. Thus, patients who received percutaneous drainage with or without ERCP (3% and 7%, respectively) were included in the meta-analysis when no additional data were available after contacting the authors (12,21). In the 9 studies that reported the incidence of failed ERCP (median = 3.3%, range 0%–9.9%), all but one study considered the first ERCP as the index procedure to quantify the time to ERCP (5,6,10,12–15,17,18,20). One study included 4 of 166 patients (2.4%) as having a history of failed ERCP, and the repeat ERCP was chosen as the index procedure to calculate the time to ERCP (10). In 2 large studies (N = 77,323 and 4,570) extracting US claims data, physician billing codes were used to identify patients with acute cholangitis, and one study defined the time to ERCP by day 0 of hospitalization as ERCP performed <24 hours, day 0–1 as ERCP performed <48 hours, and days 2–7 as ERCP performed >48 hours (16). The impact of ERCP performed <12 hours from hospitalization on mortality was evaluated in 2 studies (17,19), <24 hours in 9 studies (5,6,10,11,13,14,17,18,21), <48 hours in 8 studies (5,6,11,13,14,16,18,21), and <72 hours in 4 studies (5,6,11,14). Furthermore, the impact of ERCP performed <12 hours from hospitalization on hospital stay was evaluated in 3 studies (5,17,19), <24 hours in 10 studies (5,6,10–14,17,20,21), <48 hours in 7 studies (5,6,11,13,14,16,21), and <72 hours in 4 studies (5,6,11,14). Other outcomes data, including ICU admission, were provided in 3 studies (5,10,20), organ failure in 2 studies (10,21), ICU stay in 2 studies (5,21), 30-day readmission in 2 studies (15,16), and adverse events in 2 studies (10,17).

### Quality assessment of the included studies

All 14 studies were observational studies, including 13 retrospective studies and one prospective study. No controlled studies have been conducted to evaluate the impact of the optimal timing of ERCP on any outcomes in acute cholangitis. The median Newcastle–Ottawa Scale was 7 (range 6–8).

### Mortality in cholangitis

In 14 studies (N = 84,063), the mean age of hospitalized patients with acute cholangitis was 66 ± 18 years, and 38,686 (46%) were men. The overall pooled in-hospital mortality was 1.9% (95% CI 1.8%–7.6%). When we excluded the 2 studies that used large US administrative data, the remaining studies (N = 2,170) demonstrated pooled in-hospital mortality of 4.3% (95% CI 1.8%–8.7%). In 3 studies, including one without published result, all showed no association between acute pancreatitis and mortality (5,6,19).

### ERCP performed <24 hours on mortality

**Figure 2.**
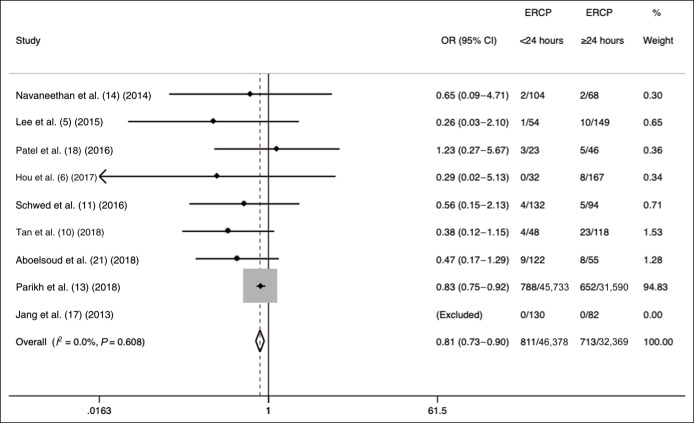
Effects of ERCP performed <24 vs ≥24 hours on mortality in acute cholangitis. ERCP, endoscopic retrograde cholangiopancreatography.

In 9 studies, ERCP was performed <24 hours from presentation in 46,367 of 78,747 patients with cholangitis (59%; 95% CI 34%–60%). In 6 studies (5,10,13,17,18,21)with available data, the mean age of patients receiving ERCP <24 hours (MD = −0.6 years, 95% CI −4.2 to 3.0, *I*^2^ = 82.3%) was not different from those who received ERCP >24 hours from presentation. ERCP performed <24 hours was associated with lower in-hospital mortality (OR = 0.81, 95% CI 0.73–0.90; *I*^2^ = 0%) compared with that performed ≥24 hours after the initial presentation (Figure 2). The funnel plots suggested possible publication bias (Begg test *P* = 0.90; Egger test *P* = 0.04). In the subgroup analyses, ERCP performed <24 hours was associated with lower in-hospital mortality (OR = 0.48, 95% CI 0.28–0.82; *I*^2^ = 0%) compared with that performed ≥24 hours after the initial presentation, even after excluding a large US administrative data study (13). Furthermore, in US studies, ERCP performed <24 hours was associated with decreased in-hospital mortality compared with that performed ≥24 hours (OR = 0.82, 95% CI 0.74–0.91; *I*^2^ = 0%). Finally, ERCP performed <24 hours was associated with decreased in-hospital mortality with acute cholangitis defined by the Tokyo Guidelines (OR = 0.52, 95% CI 0.29–0.94; *I*^2^ = 0%) or other criteria (OR = 0.83, 95% CI 0.74–0.92; *I*^2^ = 0%).

### ERCP performed <12, 48, and 72 hours on mortality

**Figure 3.**
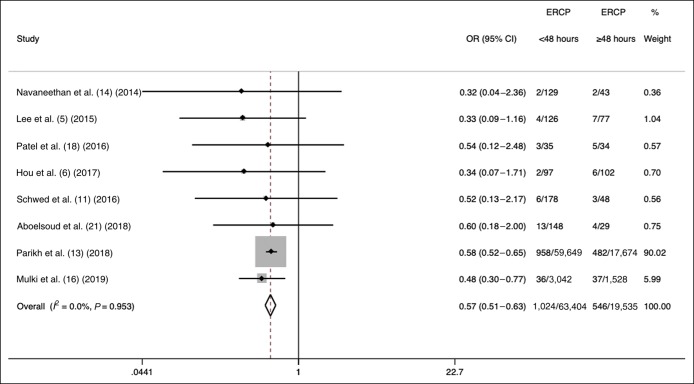
Effects of ERCP performed <48 vs ≥48 hours on mortality in acute cholangitis. ERCP, endoscopic retrograde cholangiopancreatography.

**Figure 4.**
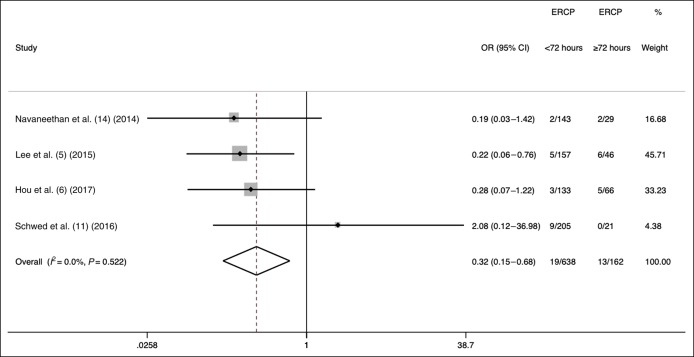
Effects of ERCP performed <72 vs ≥72 hours on mortality in acute cholangitis. ERCP, endoscopic retrograde cholangiopancreatography.

In 2 studies, ERCP was performed <12 hours from the initial presentation in 301 of 452 patients with mild-to-moderate cholangitis (67%). There was only one death in this group of patients. In 8 studies, ERCP was performed <48 hours from presentation in 63,404 of 82,939 patients (76.4%; 95% CI 56.9%–78.8%). ERCP performed <48 hours decreased in-hospital mortality compared with that performed ≥48 hours from presentation (OR = 0.57, 95% CI 0.51–0.63; *I*^2^ = 0%) (Figure 3). Furthermore, ERCP performed <48 hours decreased in-hospital mortality (OR = 0.47, 95% CI 0.32–0.67; *I*^2^ = 0%) compared with that performed ≥48 hours from presentation, even after excluding 2 studies that used large US administrative data. In 4 studies, ERCP was performed <72 hours from presentation in 638 of 800 patients (79.8%; 95% CI 63.5%–95.5%). ERCP performed <72 hours decreased in-hospital mortality compared with that performed ≥72 hours from presentation (OR = 0.32, 95% CI 0.15–0.68; *I*^2^ = 0%) (Figure 4).

### Timing of ERCP on hospital stay

In 10 studies, patients receiving ERCP <24 hours from presentation had reduced hospital stay as compared with those who received ERCP ≥24 hours (MD = 3.2 days, 95% CI 2.3–4.1; *I*^2^ = 78%) with significant heterogeneity. In 7 studies, patients receiving ERCP <48 hours showed reduced hospital stay as compared with those who received ERCP ≥48 hours (MD = 3.6 days, 95% CI 2.1–5.1; *I*^2^ = 98%) with significant heterogeneity. In 4 studies, patients receiving ERCP <72 hours compared with those who received ERCP ≥72 hours (MD = 4.1 days, 95% CI 0.9–7.3; *I*^2^ = 63%) was associated with reduced hospital stay. The sensitivity analysis demonstrated that no single study affected these results.

### Other outcomes

Other outcomes when performing ERCP <24 and 48 hours from presentation, including receiving intensive care, ICU stay, organ failure, adverse events, and 30-day readmission, were evaluated in 2 or 3 studies. In 3 studies (5,10,20), no difference (OR = 1.01, 95% CI 0.59–1.72; *I*^2^ = 36.1%) in the proportion of patients receiving intensive care was observed between performing ERCP before or 24 hours after presentation. Two studies evaluated the proportion of patients with persistent organ failure (10,21) and adverse events (10,17) without difference when ERCP was performed within or 24 hours after presentation. Decreased ICU stay (5,21) (MD = 1.6 days, 95% CI 0.2–3.0; *I*^2^ = 0%) and proportion of patients with persistent organ failure (5,21) (OR = 0.51, 95% CI 0.31–0.86; *I*^2^ = 32.6%) were observed when ERCP was performed <48 hours compared with that performed ≥48 hours in 2 studies. Finally, the incidence of 30-day readmission after hospitalization for acute cholangitis was reported in 3 studies (14,16,19), ranging from 11.5% to 24.4%. Performing ERCP <48 hours was associated with decreased odds of 30-day readmission (OR = 0.60, 95% CI 0.50–0.72; *I*^2^ = 0%) in 2 studies (15,16).

## DISCUSSION

We conducted a systematic review and meta-analysis to evaluate the optimal timing of ERCP in hospitalized patients with acute cholangitis. Our results demonstrated pooled in-hospital mortality of 1.9% (95% CI 1.8%–7.6%) in all studies and 4.4% (95% CI 1.8%–8.7%) when administrative database studies were excluded. ERCP performed <24 hours compared with ≥24 hours from presentation was associated with decreased in-hospital mortality (OR = 0.81, 95% CI 0.73–0.90). Similarly, ERCP performed <48 hours had reduced mortality compared with that performed ≥48 hours (OR = 0.57, 95% CI 0.51–0.63), and ERCP performed <72 hours had reduced mortality as compared with that performed ≥72 hours after the initial presentation (OR = 0.32, 95% CI 0.15–0.68). Finally, hospital stay was reduced when ERCP was performed <24 hours compared with ≥24 hours, <48 hours compared with ≥48 hours, and <72 hours compared with ≥72 hours with significant study heterogeneity.

Severe acute cholangitis is associated with high mortality, and biliary drainage with ERCP is a life-saving intervention. However, technical demand and resource‐intensive nature of performing urgent ERCP challenge the management of cholangitis in clinical practice. Previous studies demonstrated that early compared with the delayed performance of ERCP reduces persistent organ failure, intensive care admission and stay, and early 30-day readmission (4–6,15). However, given the infrequent incidence of acute cholangitis, previous individual studies have failed to demonstrate the impact of early ERCP on mortality with insufficient sample size. The Tokyo Guidelines 2018 recommend urgent biliary drainage for moderate or severe cholangitis, without a specified time frame (7). The 2019 European Society of Gastrointestinal Endoscopy guideline recommends ERCP <48–72 hours for moderate and <12 hours from presentation for severe acute cholangitis with low level of evidence (8).

The pooled in-hospital mortality of 1.9% (95% CI 1.8%–7.6%) was lower compared with older studies that include surgical biliary drainage, ranging 11–21% (22,23). The pooled in-hospital mortality was higher at 4.3% (95% CI 1.8%–8.7%) when the 2 largest studies (N = 77,232 and 4,570) using US administrative data were excluded. Although it is possible that mortality rate has decreased over time, reflective of increased use of ERCP as the primary therapy and improved technical success (24,25), the lower mortality rate may also be explained by the differences in study design. The limitations of administrative studies include susceptibility to inaccuracy and bias by physician coding practices. Given that the gold standard of acute cholangitis does not exist, less rigorous definition of cholangitis compared with other studies may have affected the results. In addition, the population with acute cholangitis secondary to choledocholithiasis in one study may have lowered the mortality rate (1,13). A contemporaneous study of 6,188 hospitalized patients with acute cholangitis receiving biliary drainage from Taiwan and Japan between 2011 and 2012 demonstrated all-cause 30-day mortality of 4.6% similar to 4.3% in the analysis excluding studies using administrative data from our study (1). In a portion of patients with concomitant acute cholangitis and acute pancreatitis, limited evidence demonstrated no increased risk of inpatient mortality. Although a sensitivity or subgroup analysis was not possible, acute pancreatitis is likely not a primary driver of mortality in this population. A recent study demonstrated modest inpatient mortality of 2% in 95 patients with concomitant acute cholangitis and acute pancreatitis receiving ERCP (26). Furthermore, another study reported no elevated risk of mortality in 32 patients with concomitant acute cholangitis and acute pancreatitis compared with 87 patients with acute cholangitis alone (27).

The results of our meta-analysis demonstrated that early ERCP is associated with reduced mortality in patients with acute cholangitis at all cutoff points: <24, <48, and <72 hours. Importantly, urgent ERCP performed <24 hours compared with ≥24 hours from presentation demonstrated an approximately 20% reduction in in-hospital mortality. In a subgroup analysis, excluding a large administrative data study (N = 77,323) comprising 95% of meta-analysis weight, there was more than 50% reduction in mortality when ERCP was performed <24 compared with ≥24 hours (13). Our findings are consistent with a Denmark study of 166 patients with acute cholangitis demonstrating that the performance of ERCP <24 hours was associated with lower 30-day mortality (adjusted OR = 0.23, 95% CI 0.05–0.95) after adjusting for other factors (10). Although a meta-analysis comparing ERCP performed <12 and ≥12 hours from presentation was not possible in our study, a previous study demonstrated that ERCP performed even earlier at <12 hours compared with ≥12 hours from the onset of shock was associated with decreased mortality (24). Finally, another study showed that each day of delay in the performance of ERCP was associated with a 17% (95% CI 5%–29%) relative risk increase in persistent organ failure as a surrogate endpoint of mortality regardless of cutoff times (5). In addition to the decreased in-hospital mortality with performing urgent ERCP <24 hours from presentation, lower ORs of performing ERCP <72 and <48 hours compared with <24 hours in our study underscore the deleterious effects of delay on the performance of ERCP. For example, a delay of ERCP of ≥72 compared with <72 hours from presentation translated to more than a threefold increase in the risk of inpatient mortality.

Parallel to the findings of reduction of in-hospital mortality with earlier ERCP, the meta-analyses demonstrated that ERCP performed <24 hours compared with ≥24 hours (MD = 3.2 days, 95% CI 2.3–4.1; *I*^2^ = 78%), <48 hours compared with ≥48 hours (MD = 3.6 days, 95% CI 2.1–5.1; *I*^2^ = 98%), and <72 hours compared with ≥72 hours (MD = 4.1 days, 95% CI 0.9–7.3; *I*^2^ = 63%) were associated with a reduction in hospital stay. Although the presence of study heterogeneity warrants caution in the interpretation of the findings, the mean reduction in hospital stay of >3 days with the performance of ERCP <24, <48, and <72 hours from presentation suggests the impact of early ERCP on the clinical course of acute cholangitis other than simply reducing hospital days from performing an earlier procedure. Previous studies in patients receiving ERCP for acute cholangitis showed that a procedural delay resulted in increased hospital stay as a function of time (8,14).

Our findings have clinical implications. Implementation of performing early ERCP (i.e., <24 hours from presentation) will present substantial barriers and resource strains in clinical practice because of a number of reasons. First, early diagnosis of acute cholangitis is not straightforward. In the absence of a specific biomarker, a high-level index of suspicion by the first-line healthcare provider, generally by the emergency department physician, is paramount. Given the frequent nonspecific presentation, the Tokyo Guidelines 2018 propose a diagnostic criterion (meeting 2 or 3 conditions: systemic inflammation, cholestasis, and imaging findings) to assist the identification of patients with suspected or diagnostic for acute cholangitis (28). Second, even after the diagnosis of acute cholangitis is established, appropriate triage to stratify the severity of acute cholangitis is important. Although the same guideline proposes a criterion to grade the severity, application in clinical practice may be difficult given the infrequent incidence of acute cholangitis and the complexity of the current risk stratification tool (26). Third, timely coordination with multiple disciplines (emergency department physician, gastroenterologist, anesthesiologist, and intensivist) and GI laboratory are required to facilitate an urgent procedure, which will inevitably disrupt other routine care. Furthermore, given the technical complexity, increased risk of adverse events, and frequent clinical instability in patients with acute cholangitis, coordination to perform anesthesia-assisted ERCP may further delay care (4,20). In 2 US studies, ERCP was performed with anesthesia support in 11% and 54% of the procedures (5,6). Finally, at the systems level, centers without ERCP capability should facilitate early transfer to referral centers, ideally from the emergency department. In centers providing ERCP services, an urgent procedure will necessitate continuous staffing of biliary endoscopists, including off-hours and weekends, similar to the management of upper GI bleeding. In the analyzed studies, mostly from the tertiary centers, the proportion of patients who received ERCP <24 hours from presentation was less than 60%, highlighting the challenges. In a study evaluating 4,570 patients receiving ERCP for acute cholangitis, a higher proportion of patients hospitalized on weekends (31% vs 20%, <0.0001) compared with weekdays was likely to have delayed ERCP (16).

Our meta-analysis has limitations. All included studies were observational studies that are susceptible to bias and confounding. Although hypothesis generating, observational studies do not confirm the cause and effect of early ERCP on outcomes. Factors other than the timing of performing ERCP, such as age, comorbidity, and anticoagulation status, may confound the effect on in-hospital mortality. In addition, a variable proportion of patients with concomitant acute pancreatitis or immunosuppression among the study populations may have introduced study heterogeneity for these factors. However, the results based on a limited number of studies suggested a lack of or minimal impact on mortality. Furthermore, most studies included in the meta-analysis did not stratify the severity of acute cholangitis, and a subgroup analysis to assess the impact of urgent ERCP on patients with the highest risk of mortality was not possible. Validating the impact of early ERCP on the high-risk population will be important for prioritizing care. The lack of randomized controlled studies, yet, robust number of observational studies, underscores the challenge of studying the impact of time-sensitive intervention on a relatively infrequent disease. A well-designed multicenter study evaluating the role of urgent ERCP is needed. For example, a study that compares ERCP to be performed <24 hours vs 24–72 hours from presentation stratified by the severity of acute cholangitis may clarify the impact of the optimal timing of ERCP on outcomes (29).

In conclusion, the meta-analysis of observational studies showed that the performance of earlier ERCP, even those urgently performed <24 hours from presentation, was associated with a 20%–50% reduction in mortality. Furthermore, ERCP performed <24 hours compared with ≥24 hours resulted in an approximately 3-day reduction in hospital stay. Given the limitations of observational studies, a well-designed randomized controlled study is needed to evaluate the impact of urgent ERCP on outcomes.

## CONFLICTS OF INTEREST

**Guarantor of the article:** John J. Kim, MD, MS.

**Specific author contributions:** L.D. and J.J.K.: planning and conducting the study, collecting and/or interpreting data, and/or drafting the manuscript. M.C.: collecting and/or interpreting data. X.Z., L.L., and A.S.: drafting the manuscript.

**Financial support:** This study was funded by the National Natural Science Foundation of China (no. 81800476), Zhejiang Provincial Natural Science Foundation (Q18H030013), and Zhejiang Provincial Medical Health Science and Technology Projects (grant nos. 2018254219, 2019322028, and 2018KY112).

**Potential competing interests:** None to report.
